# Criteria for item selection for a preference-based measure for use in economic evaluation

**DOI:** 10.1007/s11136-020-02718-9

**Published:** 2020-12-07

**Authors:** Tessa Peasgood, Clara Mukuria, Jill Carlton, Janice Connell, John Brazier

**Affiliations:** 1 School of Health and Related Research, University of Sheffield, Sheffield, UK; 2 Melbourne School of Population and Global Health, University of Melbourne, Melbourne, VIC Australia

**Keywords:** PROMs, Item selection, Question selection, Preference-based measures, Quality of life, Utility

## Abstract

Preference-based measures allow patients to report their level of health, and the responses are then scored using preference weights from a representative general population sample for use in cost utility analysis. The development process of new preference-based measures should ensure that valid items are selected to reflect the constructs of interest included in the measure and that are suitable for use in preference-elicitation exercises. Existing criteria on patient-reported outcome measures (PROMs) development were reviewed, and additional considerations were taken into account in order to generate criteria to support development of new preference-based measures. Criteria covering 22 different aspects related to item selection for preference-based measures are presented. These include criteria related to how items are phrased to ensure accurate completion, the coverage of items in terms of range of domains as well as focus on current outcomes and whether items are suitable for valuation. The criteria are aimed at supporting the development of new preference-based measures with discussion to ensure that even where there is conflict between criteria, issues have been considered at the item selection stage. This would minimize problems at valuation stage by harmonizing established criteria and expanding lists to reflect the unique characteristics of preference-based measures.

## Background

**Fig. 1:**
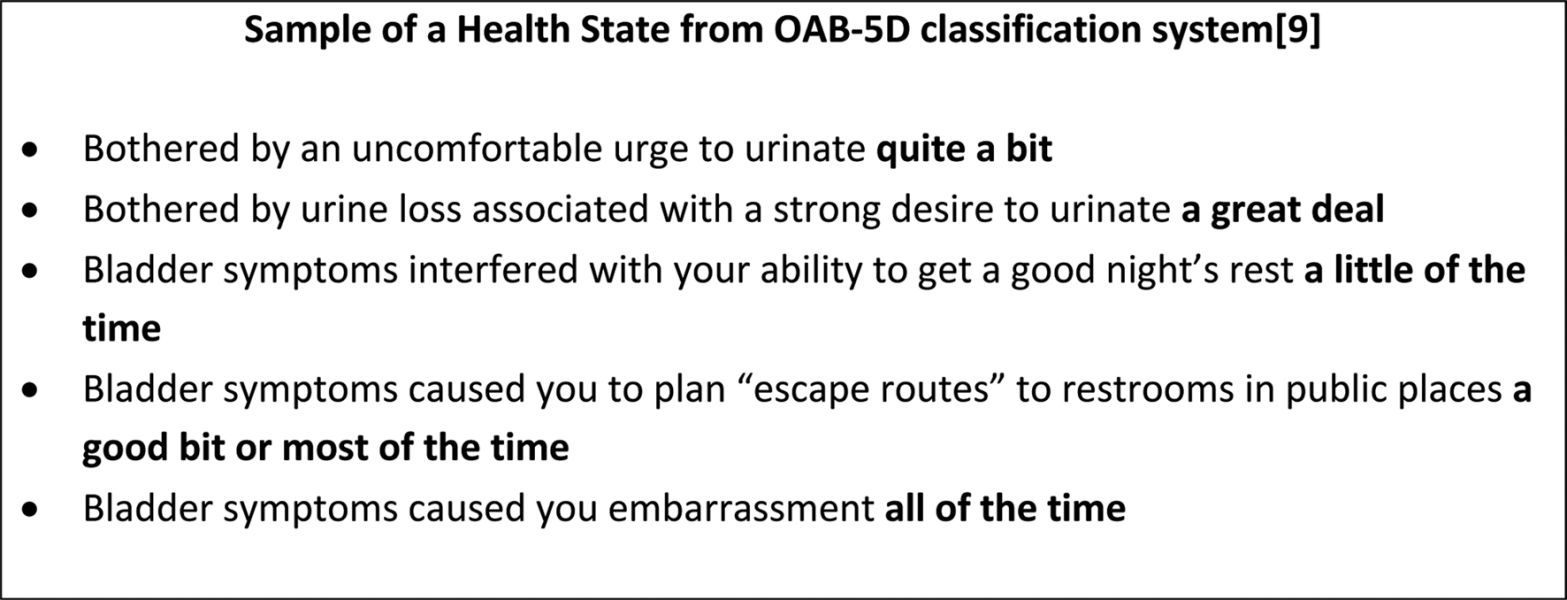
Sample of a Health State from OAB-5D classification system [9]

In the context of health technology assessment (HTA), reimbursement agencies such as the UK’s National Institute of Health and Care Excellence (NICE) recommend the use of quality-adjusted life years (QALYs) as the outcome measure [1]. QALYs combine length of life with health-related quality of life (HRQoL). The HRQoL score here is based on preferences which are anchored on a scale of dead (0) to full health or full health-related quality of life (1). These quality adjustment values are based on individuals’ preferences for different health states using preference-elicitation or valuation techniques such as time trade-off (TTO) or discrete choice experiments (DCE) [2] which aim to measure how good respondents think it would be to live hypothetical lives. Although these quality adjustment values can be generated for each individual study, this would be costly and time consuming. Preference-based measures [2] have been developed to allow patients to report their level of health, and the measure is then scored using preference weights from a representative general population sample. For preference-based measures, generating these quality adjustment values is, therefore, part of the development process. Items (or a selection of the items) are used to describe a set of health states that are then valued and used to model the quality adjustment values for all the health states described by the measure. During preference-elicitation tasks, the state to be valued is usually presented as a list of phrases which integrate the response option for each item (see Fig. 1 for an example state presented during the preference-elicitation exercise for valuing the overactive bladder preference-based measure, the OAB-5D [3]). The respondent will be asked to imagine living in the state described by these phrases.

There are rarely more than 8–9 items included in preference-elicitation tasks, and each domain is normally represented by a small number of items (usually only one). The overall number of items is limited by how much information a respondent can take in during a preference-elicitation task. The need to use the items in valuation exercises brings additional considerations relating to item suitability. The aim of this paper is to describe these additional considerations and provide a set of criteria for item selection from the perspective of developing a preference-based measure.

## Item selection criteria for a measure of QoL which will be valued based on public preferences

Item selection occurs after the conceptual framework for a new measure has been established. For preference-based measures, the conceptual framework identifies independent domains that require one (or potentially more) questions that will be used in valuation. This differs from profile measures which may have several questions representing each domain. There is also the additional complexity that although patients complete the preference-based measure, the valuation is often undertaken by members of the public who may or may not have any health condition. This requires consideration of the criteria in relation to both those completing measures *and* those undertaking the valuation. In this article, we consider how standard item selection criteria should be modified and discuss some additional criteria which could usefully be considered to ensure items will be appropriate to take forward to valuation. The list has been derived from existing lists [4–8] and through presentations and discussion with researchers and advisors associated with the ‘Extending the QALY’ project (https://scharr.dept.shef.ac.uk/e-qaly/). This article has arisen out of a project to develop a new instrument (the EQ-HWB) to capture the impact of interventions on patients, carers and social-care users (hence capturing health, carer and social-care^1^ related quality of life), which could be used to derive the quality adjustment value of a state to estimate QALYs.

The full set of criteria are presented in Table 1. Whilst these criteria may apply more generally to questionnaire development, our interest and the focus of our discussion are around how the criteria support item selection for preference-based measures. Criteria 1 to 12 are universal to good questionnaire design, criteria 13–19 may apply to non-preference-based measures although have greater importance for the design of preference-based measures, and criteria 20–22 relate specifically to requirements for valuation. The relevance of each criteria for a new questionnaire, and whether there is adequate evidence to suggest items meet each criteria requires discussion across development teams and with stakeholders. It is also applied iteratively across the different stages of item development and selection.

**Table 1:** Criteria for item selection

No	Criteria	Explanation
*Criteria for full and accurate completion of questions*		
1	Reading level should be appropriate	The item should be easy to read and understand
2	Avoid questions that are very long	Items should be as short as possible whilst maintaining comprehensibility
3	Avoid double negative	Avoid phrases in which a negated construct (e.g. no control, not coping) requires a negative answer (e.g. none of the time)
4	Avoid ambiguity	Avoid questions which have a potentially ambiguous interpretation
5	Avoid jargon	The vocabulary throughout (including any labelling linked to items) should not be technical
6	Avoid terms that are colloquial	Excessively colloquial language may not be understood by all respondents and may be hard to translate to other languages
7	Avoid asking a combination of two or more questions within one item	Where items contain two or more questions at the same time (e.g. anxiety or depression), it is not clear which the respondent is answering. Multiple terms tapping into the same construct may sometimes be required to improve comprehension
8	Avoid excessively personal questions	Excessively personal or intrusive items may lead to missing values or annoy responders
9.	Avoid ethically inappropriate questions	Consideration should be given to the appropriateness of asking items for potentially vulnerable sub-groups. Questions which might leave people in a worse frame of mind after completion should only be used where no good alternative is available
10	Avoid questions that are not relevant to all responders	Avoid items that refer to circumstances, situation or lifestyle that may not be universal across all responders
11	Avoid items that draw on knowledge beyond the individual’s experience	Items that relate to another piece of knowledge, such as what other people think, may be difficult to complete if the responder is not confident in that knowledge
12	Avoid value laden terms	Items which are value laden may lead people to believe there is a right or wrong way of answering the question
*Criteria to ensure items cover the full range of the domain*		
13	Avoid items that are too extreme or too mild	Items that only tap onto the severe or the mild end of a domain would need to be supplemented by other items on that sub-domain
*Criteria to ensure items tap into the current QoL and can be compared between and within people*		
14	Avoid items that suffer from Differential Item Functioning (DIF)	DIF identifies sub-groups of people who, despite having the same underlying level of an attribute (or latent construct), answer an item differently
15	Avoid items that make comparisons to other people	Items that make comparisons to other people depend upon whom the individual chooses to use for a comparison
16	Avoid items that make comparisons to expectations	Items which make comparisons to a person’s expectations or personal norms are problematic due to lack of inter-personal comparability of responses
17	Avoid items that make comparisons over time	Items which ask the respondent to make a comparison to another time period or to ‘usual’ are not suitable as they depend upon what the past or ‘usual’ is like for the individual
18	Avoid items that do not lend themselves to the specified time period	Items need to clearly tap into the current situation as restricted by the time period given within the questionnaire
19	Avoid items with response options that do not have clear order	Items, and underlying constructs, should have clear order, such that a better response is judged as better quality of life
*Criteria to help select items are suitable for valuation*		
20	In preference-elicitation exercises, respondents do trade-off between improvement in the item vs. improvement in another domain	All items included within a classified for valuation should be domains of life that are important
21	The item does not attribute	Avoid items where a decrement or problem is attributed to a particular circumstance
22	Response options are in the same direction	Within the description of the state for valuation, responders to valuation exercises are likely to get confused if ‘often’ feeling X is sometimes a good thing and sometimes a bad thing

### Accuracy and completion

Criteria 1–12 relate to accurate and complete responses and aiming for questions which are “brief, clearly worded, easily understood, unambiguous and easy to respond to” [4].

The first three relate to ensuring items are easy to read. Steiner and Norman [5] recommend a reading age of not more than 12 years for patient-reported outcome measures (PROMs) [9]. Reading ease can be assessed by looking at some combination of number of words per sentence, number of syllables per word, ratio of complex words to easy words and number of characters per word (e.g. Flesch Kincaid Grade Level, Gunning Fog Score, SMOG Index, Coleman Liau Index, ARI). Many of these reading level assessments focus upon whole blocks of text, but for preference-elicitation tasks, the item will be read alone and out of context hence offering less contextual clues for the individual to draw upon to aid reading. This implies a need for reading level of isolated items to be evaluated (sensitively) within qualitative work.

Bradburn et al. [8] notes that ill patients and some older people in particular may be confused by long complicated sentences, suggesting a need to keep items short whenever possible (Criteria 2). When response options are built into the question through repeating a core question stem (e.g. I had no pain, I had slight pain, I had moderate pain etc.) respondents may get frustrated at being asked to read repetitive text. However, when the endorsed statement is used within a health state classification (see Fig. 1), the built in style has an advantage in that it enables the exact wording to be shown within the description of the state to be valued – therefore, what is valued is also what is described by the full measure.

Double negatives [8] make the items difficult to understand and to complete (Criteria 3). Double negatives may be created by the choice of response options, e.g. *I felt I had no control – none of the time*. When respondents are completing measures, the full set of options will be visible, e.g. for frequency options (most of the time, often, occasionally etc.) and the double negative may be less problematic. However, once the state is described for valuation, the appearance of the item makes this double negative clash more apparent.

Criteria 4 seeks to avoid items which are potentially ambiguous because they are too complex, vague, hard to interpret, or could be interpreted in different ways. For example, a term such as ‘*satisfied’*’ may be problematic as it can be interpreted as a positive or a neutral state. Ambiguity also arises where the individual’s specific context or circumstances impact upon their interpretation of items, for example, ‘*being able to communicate’*’ may relate to social media use for some young people, presentation skills for some working age individuals, or the ability to be understood when speaking for post-stroke patients.

Avoiding specialist terminology or jargon (Criteria 5) also relates to ambiguity since some respondents may interpret the meaning of an item in its specialist sense and others may not – or may not understand it at all. This includes medical terminology where some respondents may link to specific meanings or diagnostic criteria and others may apply a more colloquial interpretation. For example, if asked about ‘*being depressed’*’ some respondents may only respond positively if they have a diagnosis of depression. Colloquial items (Criteria 6) such as ‘*feeling down in the dumps’*’ are also likely to be problematic for consistent interpretation, translation, and may date quickly.

Respondents may find it difficult to answer items where two or more questions are asked at the same time [5] (Criteria 7). For the patient completing the questionnaire, there may be a conflict between the different components of an item. It is also problematic for valuation as those undertaking valuation may focus on one part of the question, e.g. the SF-6D item ‘You feel tense or downhearted and low’ [10]. In valuation of the EQ-5D item on pain/discomfort (*I have moderate pain or discomfort*), valuation has been found to focus mainly on pain [11].

Some questions use two or more component parts to help clarify the meaning of a single construct. For example, the question ‘*I was able to focus and concentrate*’, is asking about the same domain. Whilst this may help convey clarity of meaning, it may still raise problems if part of the compound taps into mild problems and the other taps into more severe problems. It will not then be clear which level of severity should be focused upon in the valuation. There may be a trade-off between this criteria and also needing to adequately communicate the concept of interest, which may be best done through the use of additional terms.

The next three criteria promote high completion rates across all relevant groups. Highly personal or intrusive items (Criteria 8), such as suicide ideation or problems with sexual activity, may lead to missing values or annoy or upset responders. Bradburn et al. [8] notes that potentially embarrassing or offending questions, if necessary, should be included at the end with an opt-out condition used. However, algorithms to generate a quality adjustment value require complete data across all items; hence, missing data should be avoided.

Consideration needs to be given to the ethical issues around asking specific questions to vulnerable groups (Criteria 9), for example, individuals caring for very sick or dying loved ones, individuals with severe physical and mental health problems, or individuals with very limited remaining life expectancy. How completing the question is likely to make people feel is a legitimate concern at the item selection stage. Asking positively framed questions (e.g. how satisfied are you with your life?) may be insensitive for those in very difficult circumstances or for those close to the end of life. Asking particularly negative questions (e.g. I felt like a failure) may spark upsetting feelings or thoughts for responders. Asking questions relating to safeguarding concerns (such as suicide ideation) may be problematic where no clinical follow-up is incorporated.

Criteria 10 relates to avoiding items that refer to a particular circumstance, situation or lifestyle that may not be universal across all future responders. This includes avoiding questions which refer to spouses or families; refer to employment; refer to particular activities or circumstances which might not be relevant to all (e.g. questions about working or sexual activity). If the domain is not relevant, this may result in missing data, which as noted, would make it difficult to generate the quality adjustment value for the state.

Criteria 11 recommends that items do not draw upon another piece of knowledge, such as what other people think, as this may be difficult to complete if the responder is not confident in that knowledge, e.g. ‘*other people care about me*’, ‘*I am a burden to others*’. This also has the problem (discussed below) of attribution – in valuation it might be the actual burden to others which is valued or the experience for the individual of feeling like a burden.

Criteria 12 recommends caution around potentially value laden or judgmental questions which may lead to socially desirable responding [12]. The tone of the question should be neutral to avoid respondents trying to conform to social norms or present themselves in a good light. That said, many instruments may draw on a theory of quality of life which does contain normative judgments. For example, a question on extent of social contact may be included on the basis that more social contact is assumed to be an improvement in QoL (even though some people may not agree with this). Normative judgments within a measure should be clear and transparent and not arise accidentally through choice of item.

### Ensure items cover the full range of the domain

The next criteria (Criteria 13) relates to coverage across domains of interest which is particularly relevant in preference-based measures where few items (often just one) are used to represent domains. An item such as ‘*I have problems feeding myself*’ may not be sufficient on its own to identify personal-care limitations across the full domain or latent construct. Item response theory (IRT) analysis can illuminate where an item provides information across the latent construct, hence, enabling selection of items which provide accurate information across the full range of the construct. There will be a trade-off between including more items to enable greater precision across the full range of the latent construct and overall length. Given the aim of supporting economic evaluation of (usually publicly funded) interventions, the final instrument needs to be sensitive to alleviation of suffering of those receiving care – hence, items should provide information at the end of the latent construct representing poor QoL.

### Ensure items are suitable for measuring QALYs

Criteria 14–19 relate to the need to avoid items that will be unsuitable for estimating the quality adjustment component used in the calculation of QALYs. QALY calculations require an assumption that states can be valued independently to their duration or their position within a sequence of states [13]. The quality adjustment for a state is assumed to be inter-personally and inter-temporally comparable. Consequently, it is important that each item is clearly tapping into the specific time period and does not rely upon comparisons to other people or other time periods.

Inter-personal comparisons rely upon the absence of differential item functioning (DIF) [14] (Criteria 14) which identifies sub-groups who, despite having the same underlying level of an attribute, answer an item differently (either consistently across the domain: uniform DIF, or with a different degree of difference across the domain: non-uniform DIF). For example, crying questions can be answered differently between men and women even when they have the same level of depression [15]. DIF may arise when different groups interpret items in different ways.

Psychometric analysis can test for DIF across different groups where there is a hypothesized reason for exploring this difference (e.g. age, gender and ethnicity). One problem with QoL items arises because potentially relevant domains may also be symptoms of certain health conditions. Where items tap into specific symptoms, we may expect to see DIF for that patient group, for example, feelings of hopefulness may be part of a full QoL and general positive affect questionnaire, yet also a symptom of depression. Hence findings of DIF need to be interpreted with caution.

Inter-personal comparability also includes avoiding items that make comparisons to other people directly (Criteria 15) or to expectations (Criteria 16). Items that make comparisons to others depend upon who the individuals choose to use for a comparison, therefore, conflict with the need for inter-personal comparability, e.g. ‘*I felt just as good as other people*’ depends which ‘other people’. Where one item adopts a comparison approach and others do not, we would expect to identify DIF on that item. Given the self-complete nature of items, this may not be avoided entirely; however, items which are less likely to draw on individual expectations will be preferred.

Items which ask the respondent to make a comparison to another time period or to ‘usual’ are not suitable as they depend upon what the past or ‘usual’ is like for the individual (Criteria 17), e.g. ‘*I’m bothered by things that don’t usually bother me’’*.

Criteria 18 focuses on the need for items which clearly link to a specified time period. Items will not be suitable if they refer (directly or via the respondent’s interpretation) to the recent or distant past beyond the specified time period, or to the future. For example, an item using the term ‘life’ as in ‘*how good is your life*’ may not lend itself to a confined time period. Including a specific time period for consideration in the preamble to the question may not overcome the time-period framing created by the item. This includes items which could be interpreted as referring to a personality trait drawing outside the specified time period, e.g. ‘*I had a bad temper*’. If the items refer to the last seven days, then the respondents’ answer should be based only on their judgment of the last seven days.

Criteria 19 seeks to avoid items, and underlying constructs, in which there may be disagreement about whether a better response option always represents a better quality of life. This may be discussed in qualitative work. For example, people may disagree as to whether more self-confidence, control or independence is always a good thing. Quantitative assessment using IRT or Rasch analysis can also be used to assess whether response options are ordered as expected.

Criteria 20–22 relate specifically to considerations of the valuation task. All items included within a classification for valuation should be domains of life that are important, with supporting evidence from qualitative interviews or previous valuation studies. Those doing the valuation should be willing to trade-off improvements in the domain against other domains (Criteria 20). This criteria may be hard to establish in advance of conducting valuation exercises, reinforcing the need for an iterative approach to the design of the measure.

Trade-offs which involve more than one person may be problematic, such as the concern for others wellbeing. For example, the AQol-8D item “How much of a burden do you feel you are to other people?” [16]. If improvement in that item is traded against deterioration in another item, it will not be clear whether the individual is valuing the feeling or experience of perceiving one is being a burden, or whether they are valuing the actual burden or impact upon others.

Within a valuation tasks, it should be clear what is being valued. Items which attribute a decrement or problem to a particular circumstance will be problematic in this regard (Criteria 21). For example, an item such as “because of X I am unable to do Y” “because of my pain I am unable to see my friends”, is difficult to value due to uncertainty as to whether X (pain) or Y (seeing friends) is being valued. It is also problematic because respondents may not be able to accurately attribute the cause.

Criteria 22 relates to the direction of the framing of an item and its response options. Whether items should be positively or negatively framed has been extensively debated [17]. Positively framed items can have advantages in terms of willingness of completion, and how completing the questionnaire may impact on mood. However, this is not universal as some groups with very poor mental health or life circumstances may prefer to complete negatively framed items.

A case has been made for including both, in part to ensure those responding as ‘flat liners’ down one end of the scale would have less impact upon the average results as the positive and negative scoring would cancel each other out. However, this has been found to have a negative impact upon validity [17–19]. When items are designed for preference-elicitation tasks, it would be better for the use of response options to be in a consistent direction. Switching the meaning of terms would be confusing for respondents (e.g. where ‘none of the time’ is good (pain – a negative item) and bad (energy – a positive item)) which may have an impact on quality adjustment values.

## Other considerations

The issue of translatability of items is also important both for international use and within country use (for use in translation with multi-lingual populations and to highlight potential problems for those speaking the dominant language as a second language). There are good practice guidelines for formal translation [20]; however, each of the above criteria also needs consideration in the context of the translated version and the different cultural context – much of which can only be adequately addressed with qualitative interviews to ensure the consistent interpretation of the content of the question rather just an accurate literal translation.

## Conclusion

Many of the criteria within this checklist are well established. However, we have reflected specifically on the additional concerns around identifying appropriate items for preference-based measures which will be scored on a QALY scale. For this, individuals will be asked to trade-off improvements across different items in a classification, and between improvements in items and length of life. Both this task and subsequent assumptions placed on the interpretation of the value of the state derived from the preference-elicitation exercise bring additional constraints on appropriate items.

Meeting these criteria at the stage of item selection will limit the potential for problems arising at the stage of valuation. We recognize that these criteria may at times be in conflict and the instrument development process will need to make judgments that should be informed by patients and others who will be completing the questionnaire. Therefore, application of the criteria requires careful consideration of evidence drawn from a variety of sources including qualitative studies with those likely to complete the questionnaire and those from whom preferences will be elicited.

## Footnotes

1 Social care is the term used in the UK and some other countries to mean personal care and support to people who are disabled, elderly or severely ill.
